# Identifying translational science within the triangle of biomedicine

**DOI:** 10.1186/1479-5876-11-126

**Published:** 2013-05-24

**Authors:** Griffin M Weber

**Affiliations:** 1 Harvard Medical School, Boston, MA, USA; 2 Beth Israel Deaconess Medical Center, Boston, MA, USA; 3 Information Technology, 20 Overland St, Boston, MA, 02215, USA

**Keywords:** Translational science, Bibliometric analysis, Medical subject headings, Data visualization, Citation analysis

## Abstract

**Background:**

The National Institutes of Health (NIH) Roadmap places special emphasis on “bench-to-bedside” research, or the “translation” of basic science research into practical clinical applications. The Clinical and Translational Science Awards (CTSA) Consortium is one example of the large investments being made to develop a national infrastructure to support translational science, which involves reducing regulatory burdens, launching new educational initiatives, and forming partnerships between academia and industry. However, while numerous definitions have been suggested for translational science, including the qualitative T1-T4 classification, a consensus has not yet been reached. This makes it challenging to tract the impact of these major policy changes.

**Methods:**

In this study, we use a bibliometric approach to map PubMed articles onto a graph, called the Triangle of Biomedicine. The corners of the triangle represent research related to animals, cells and molecules, and humans; and, the position of a publication on the graph is based on its topics, as determined by its Medical Subject Headings (MeSH). We define translation as movement of a collection of articles, or the articles that cite those articles, towards the human corner.

**Results:**

The Triangle of Biomedicine provides a quantitative way of determining if an individual scientist, research organization, funding agency, or scientific field is producing results that are relevant to clinical medicine. We validate our technique using examples that have been previously described in the literature and by comparing it to prior methods of measuring translational science.

**Conclusions:**

The Triangle of Biomedicine is a novel way to identify translational science and track changes over time. This is important to policy makers in evaluating the impact of the large investments being made to accelerate translation. The Triangle of Biomedicine also provides a simple visual way of depicting this impact, which can be far more powerful than numbers alone.

## Background

In biomedicine, translational science is research that has gone from “bench” to “bedside”, resulting in applications such as drug discovery that can benefit human health [1-6]. However, this is an imprecise description; and, while numerous definitions have been suggested, including the qualitative T1-T4 classification [7], a consensus has not yet been reached. Several bibliometric techniques have been developed to quantitatively place publications along the translational spectrum. Narin assigned journals to fields, and then grouped these fields into either “Basic Research” or “Clinical Medicine” [8-10]. Narin also developed another classification called research levels, in which journals are assigned to “Clinical Observation” (Level 1), “Clinical Mix” (Level 2), “Clinical Investigation” (Level 3), or “Basic Research” (Level 4) [8]. He combines Levels 1 and 2 into “Clinical Medicine” and Levels 3 and 4 to “Biomedical Research”. Lewison showed that the research level of individual articles can be determined from keywords within the articles’ titles and addresses, and he defines the average research level of a collection of articles as the mean of the research levels of those articles [11-13].

In this study, we analyze the 20 million publications in the National Library of Medicine’s PubMed database by extending these bibliometric approaches in three ways: (1) We divide basic science into two subcategories, research done on animals or other complex organisms and research done on the cellular or molecular level. We believe it is important to make this distinction due to the rapid increase in “-omics” research and related fields in recent years. (2) We classify articles using their Medical Subject Headings (MeSH), which are assigned based on the content of the articles. Journal fields, title keywords, and addresses only approximate an article’s content. (3) We map the classification scheme onto a graphical diagram, which we call the Triangle of Biomedicine, which makes it possible to visualize patterns and identify trends over time.

## Methods

### Article classification technique

Using a simple algorithm based on an article’s MeSH descriptors, we determined whether each article in PubMed contained research related to three broad topic areas—animals and other complex organisms (A), cells and molecules (C), or humans (H). An article can have more than one topic area. Articles about both animals and cells are classified as AC, articles about both animals and humans are AH, articles about cells and humans are CH, and articles about all three are ACH. Articles that have none of these topic areas are unclassified by this method.

To determine an article’s topics, we took advantage of the fact that MeSH is organized as a hierarchical tree, and the three topic areas correspond to particular MeSH nodes and their subtrees. H is mapped to all MeSH codes under the subtrees B01.050.150.900.649.801.400.112.400.400 (Human) and M01 (Person); A is mapped to all codes under the subtree B01 (Eukaryota) except the code for Humans; and C is mapped to the subtrees A11 (Cells), B02 (Archaea), B03 (Bacteria), B04 (Viruses), G02.111.570 (Molecular Structures), and G02.149 (Chemical Processes). These mappings are not perfect. A much more complicated MeSH-based classification technique could have been developed; however, keeping the definition of the three areas simple did not seem to limit our analysis, and it made the results easier to interpret.

### The triangle of biomedicine

Several groups have created “maps of science” to visually depict the structure of literature by showing the relationships among different fields of science [14-20]. In these maps, a reference system is defined, over which data about publications and citations are placed. A reference system can be chosen specifically to highlight certain attributes of the data, such as emerging areas of innovation or interdisciplinary research.

In order to identify translational research, we constructed a trilinear graph [21], where the three topic areas are placed at the corners of an equilateral triangle, with A on the lower-left, C on the top, and H on the lower-right. The midpoints of the edges correspond to AC, AH, and CH articles, and the center of the triangle corresponds to ACH articles.

An article can be plotted on the Triangle of Biomedicine according to the MeSH descriptors that have been assigned to it. For example, if only human descriptors, and no animal or cell descriptors have been assigned to an article, then it is classified as an H article and placed at the H corner. An article with both animal and cell descriptors, and no human descriptors, is classified as an AC article and placed at the AC point. A collection of articles is represented by the average position of its articles. Although an individual article can only be mapped to one of seven points, a collection of articles can be plotted anywhere in the triangle.

An imaginary line, the Translational Axis, can be drawn from the AC point to the H corner. The position of one or more articles when projected onto this axis is the Translational Index (TI). By distorting the Triangle of Biomedicine by bringing the A and C corners together at the AC point, the entire triangle can be collapsed down along the Translational Axis to the more traditional depiction of translational science being a linear path from basic to clinical research. In other words, the Triangle of Biomedicine does not replace the traditional linear view, but rather provides additional clarity into the path research takes towards translation.

### Mathematical description of the triangle of biomedicine

The Triangle of Biomedicine is drawn as an equilateral triangle, whose corners correspond to A, C, and H topic areas. On a Cartesian system, each corner is a distance of 1 from the origin, with the A corner at (x,y) = (-sqrt(3)/2,-0.5), the C corner at (0,1), and the H corner at (sqrt(3)/2,-0.5). The AC, AH, and CH points are midway along the edges of the triangle, and the ACH point is located at the origin at (0,0). The Translational Axis is a line from the AC point, through the origin, to the H corner. The position of a point projected onto the Translational Axis is its Translational Index (TI). For example, the A, AC, or C points have TI = -0.5; the AHC point has TI = 0; the AH and CH points have TI = 0.25; and the H point has TI = 1. A collection of articles with mostly human studies that includes a small amount of basic science research will be close to the H corner, but not directly on it, and it will have a TI slightly less than 1.

### Datasets used to validate the triangle of biomedicine

Our datasets are 1) a snapshot of 20,032,189 PubMed articles and their associated MeSH descriptors and citations from December 24, 2010 (http://pubmed.org); 2) broad journal headings from the NLM Catalog database (http://www.ncbi.nlm.nih.gov/nlmcatalog); and 3) degrees and publications of 12,729 Harvard Medical School faculty taken from the Harvard Catalyst Profiles website (http://connects.catalyst.harvard.edu/profiles) in December, 2010. Each of these sources is publicly available.

### Corrected citation counts

Although we are using all PubMed articles for this study, PubMed derives its citation data (one article citing another) from PubMed Central (PMC), which represents only a subset of PubMed articles. As a result, the citation counts in PubMed are underestimates of the total number of times that articles have actually been cited. We therefore define a “corrected citation count” for an article by dividing each citation by the percentage of publications of the citing article’s type that are in PMC. For example, since 4.9% of H articles and 17.1% of C articles are in PMC, if an article has been cited in PMC by one H article and two C articles, its corrected citation count is 1/0.049 + 2/0.171 = 32.1. The assumption is that for articles of a given type, the ones in PMC cite articles the same way as the ones that are not in PMC.

Other citation databases exist, such as Thomson Reuters’ Web of Science (WoS), Elsevier’s Scopus, and Google Scholar. While there are large overlaps among these databases, there are also significant differences, which means that none of them are complete, and there will be biases regardless of which database is used [22]. We chose PMC because it is the only one that is freely available to download in its entirety, and it is linked to PubMed and MeSH.

To gain a general sense of the differences between citation databases, we compared PMC and WoS for 174,395 articles written by Harvard faculty that we identified in both databases. Table 1b compares the PMC corrected citation counts to the WoS citation counts, broken into A-C-H categories. Although the PMC corrected citation counts were higher on average than WoS (possibly due to the different distributions of articles by year in PMC and WoS), the ratios between categories were similar. For example, in both databases, ACH had the highest citation count and H had the lowest (with the exception of unclassified articles), with a ratio between ACH and H of 2.53 in PMC and 2.43 in WoS. Since the ratio determines the position on the Triangle of Biomedicine, this suggests that we would have had similar results using WoS instead of PMC.

**Table 1:** Summary of categories

**a Category**	**Number of articles**	**Percent of PubMed**	**Number of authors**	**MeSH descriptors**	**Basic research**	**Research level**
A	1,878,604	9.4%	3.42	10.42	0.634	3.15
C	826,426	4.1%	3.39	8.79	0.911	3.78
H	8,676,294	43.3%	3.24	10.02	0.125	1.59
AC	2,015,181	10.1%	3.96	13.25	0.795	3.68
AH	611,098	3.1%	3.02	10.88	0.463	2.10
CH	1,581,218	7.9%	4.68	12.60	0.562	2.85
ACH	714,372	3.6%	4.50	14.71	0.753	3.40
None	3,728,996	18.6%	2.28	2.27	0.494	2.28
**b Category**	**Percent in PMC**	**Percent cited in PMC**	**Mean PMC citations**	**Corrected PMC citations**	**Harvard corrected citations**	**Harvard WoS citations**
A	5.5	31.6	1.23	16.2	56.7	40.4
C	17.1	50.8	4.85	36.0	97.1	60.1
H	4.9	22.5	0.71	13.7	51.8	32.4
AC	13.5	54.8	4.07	37.5	112.3	67.3
AH	6.3	34.8	1.65	23.8	83.9	55.1
CH	11.3	48.2	2.99	31.7	91.5	54.7
ACH	15.2	60.1	5.48	53.1	130.8	78.7
None	7.6	12.0	0.52	7.21	32.0	34.9
**c Category**	**Translational fraction (TF)**	**Translational distance (TD)**		**Translational years (TY)**	**Translational closeness (TC)**	
A	0.198	2.46		10.40	0.107	
C	0.379	3.08		8.74	0.147	
H	0.192	1.12		5.69	0.183	
AC	0.392	2.76		8.26	0.175	
AH	0.249	1.71		6.70	0.187	
CH	0.364	1.90		6.03	0.246	
ACH	0.430	2.22		5.81	0.246	
None	0.089	2.10		13.39	0.059	

Listed for each category are (**a**) the number of articles, the percent of all articles in PubMed, the average number of authors per article, the average number of distinct MeSH descriptors that have been assigned to the articles, fraction of articles in basic research (vs basic research or clinical medicine) journals, the average Research Level; (**b**) the percent of articles in the category that exist in PubMed Central (PMC), the percent of articles that have been cited by at least one article in PMC, the mean number of times articles have been cited in PMC, the mean corrected number of PMC citations, the mean corrected number of PMC citations for a sample of articles published by Harvard researchers, the mean number of Web of Science (WoS) citations for the same sample of Harvard publications; (**c**) the Translational Fraction (TF), the Translational Distance (TD), the Translational Years (TY), and the Translational Closeness (TC).

### Mapping A-C-H categories to Narin’s basic-clinical classification scheme

The National Library of Medicine (NLM) classifies journals into different disciplines, such as microbiology, pharmacology, or neurology, with the use of Broad Journal Headings. We used Narin’s mappings to group these disciplines into basic research or clinical medicine. Individual articles were given a “basic research” score of 1 if they were in a basic research journal and 0 if they were in a “clinical medicine” journal. For each A-C-H category, a weighted average of its articles’ scores was calculated, with the weights being the inverse of the total number of basic research (4,316,495) and clinical medicine (11,689,341) articles in PubMed. That gives a numeric value for the fraction of articles within a category that are basic research, which is corrected for the fact that PubMed as a whole has a greater number of clinical medicine articles.

### Mapping A-C-H categories to Narin’s four-level classification scheme

For each of his four research levels, Narin selected a prototype journal to conduct his analyses: *The Journal of the American Medical Association* (*JAMA*, Level 1), *The New England Journal of Medicine* (*NEJM*, Level 2), *The Journal of Clinical Investigation* (*JCI*, Level 3), and *The Journal of Biological Chemistry* (*JBC*, Level 4). Each is widely considered a leading journal and has over 25,000 articles spanning more than 50 years. For each A-C-H category, we determined the number of articles from each of these four journals and calculated a weighted average of their research levels, with the weights being the inverse of the total number of articles each journal has in PubMed.

## Results

### Article classification

Table 1a lists the number of articles that map to each A-C-H category. The largest category is H, representing 43.3% of the articles in PubMed. About 19% of articles do not fit into any category, and therefore cannot be classified by this method. Many of these articles are in areas such as history of medicine and social science, and a third of them simply have no MeSH descriptors assigned to them yet.

### Comparing A-C-H categories to Narin’s classification schemes

To validate our MeSH-based classification algorithm, we used an approach similar to Lewison and compared our A-C-H categories to Narin’s two classification schemes. In both cases, our method was consistent with Narin’s:

1) If we give articles that Narin would classify as “basic research” a score of 1 and “clinical medicine” a score of 0, then H articles have a basic research score of 0.125, meaning they are mostly in clinical journals, while A and C articles have scores of 0.634 and 0.911, respectively, meaning they are mostly in basic research journals (Table 1a). AH, CH, ACH, and AC articles contain progressively more basic research, in that order.

2) Using Narin’s research levels, H articles have a score of 1.59, which is between his two Clinical Medicine levels, Clinical Observation (Level 1) and Clinical Mix (Level 2). A and C articles have research levels of 3.15 and 3.78, respectively, which are between the two Biomedical Research levels, Clinical Investigation (Level 3) and Basic Research (Level 4). AH and CH articles fall in the middle, between Levels 2 and 3; and, ACH and AC are both Biomedical Research.

### Mapping disciplines to the triangle of biomedicine

**Figure 1:**
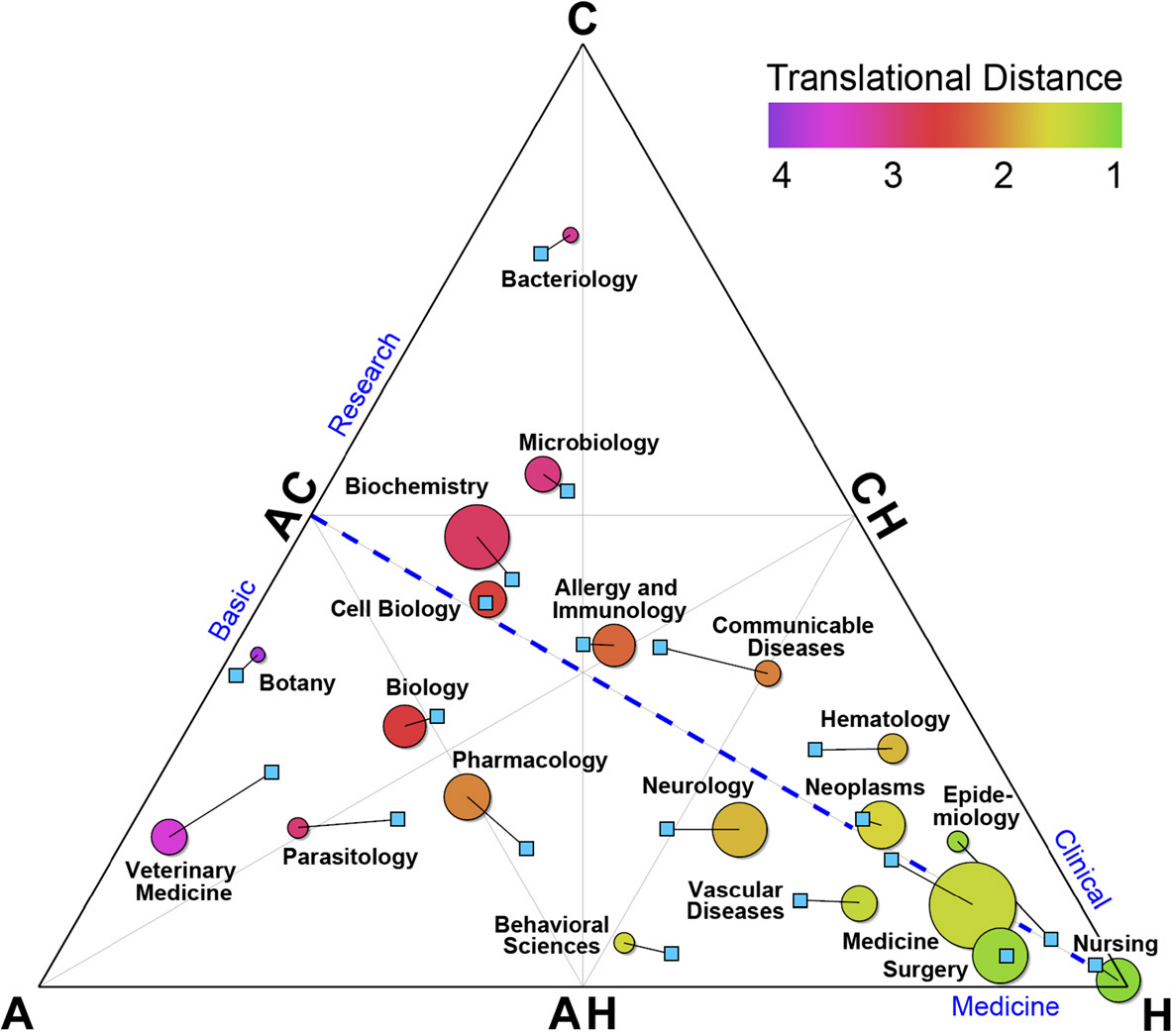
**Disciplines mapped onto the Triangle of Biomedicine. **The corners of the triangle correspond to animal (**A**), cellular or molecular (**C**), and human (**H**) research. The dashed blue line indicates the Translational Axis from basic research to clinical medicine. The position of each circle represents the average location of the articles in a discipline. The size of the circle is proportional to the number of articles in that discipline. The color of the circle indicates the Translational Distance (TD)—the average number of citation generations needed to reach an H article. The position of the light blue box connected to each discipline represents the average location of articles citing publications in that discipline. To provide clarity, not all disciplines are shown.

In Figure 1, disciplines, as defined by NLM Broad Journal Headings, are plotted onto the Triangle of Biomedicine by averaging the position of all of the individual articles in that discipline. The size of the circle is proportional to the number of articles. As one would expect, the fields closest to the A, C, and H corners are veterinary medicine, bacteriology, and nursing, respectively. Clinical specialties, such as vascular diseases and general surgery contain articles that are primarily in the H corner. Disciplines typically considered basic science, such as biochemistry and cell biology, are near the AC point. Allergy and immunology is the discipline closest to the ACH point.

The blue squares connected to each discipline indicate the average position publications that cite articles in that discipline. The angle and length of the connecting lines indicate the average direction and speed of knowledge flow. For example, articles that cite hematology studies include more animal research than the field of hematology itself, and publications that cite pharmacology or epidemiology studies include more human research. Thus, the position of a circle indicates the A-C-H composition of the research in a discipline, while the square suggests the direction of that discipline’s impact.

### Identifying changes over time

**Figure 2:**
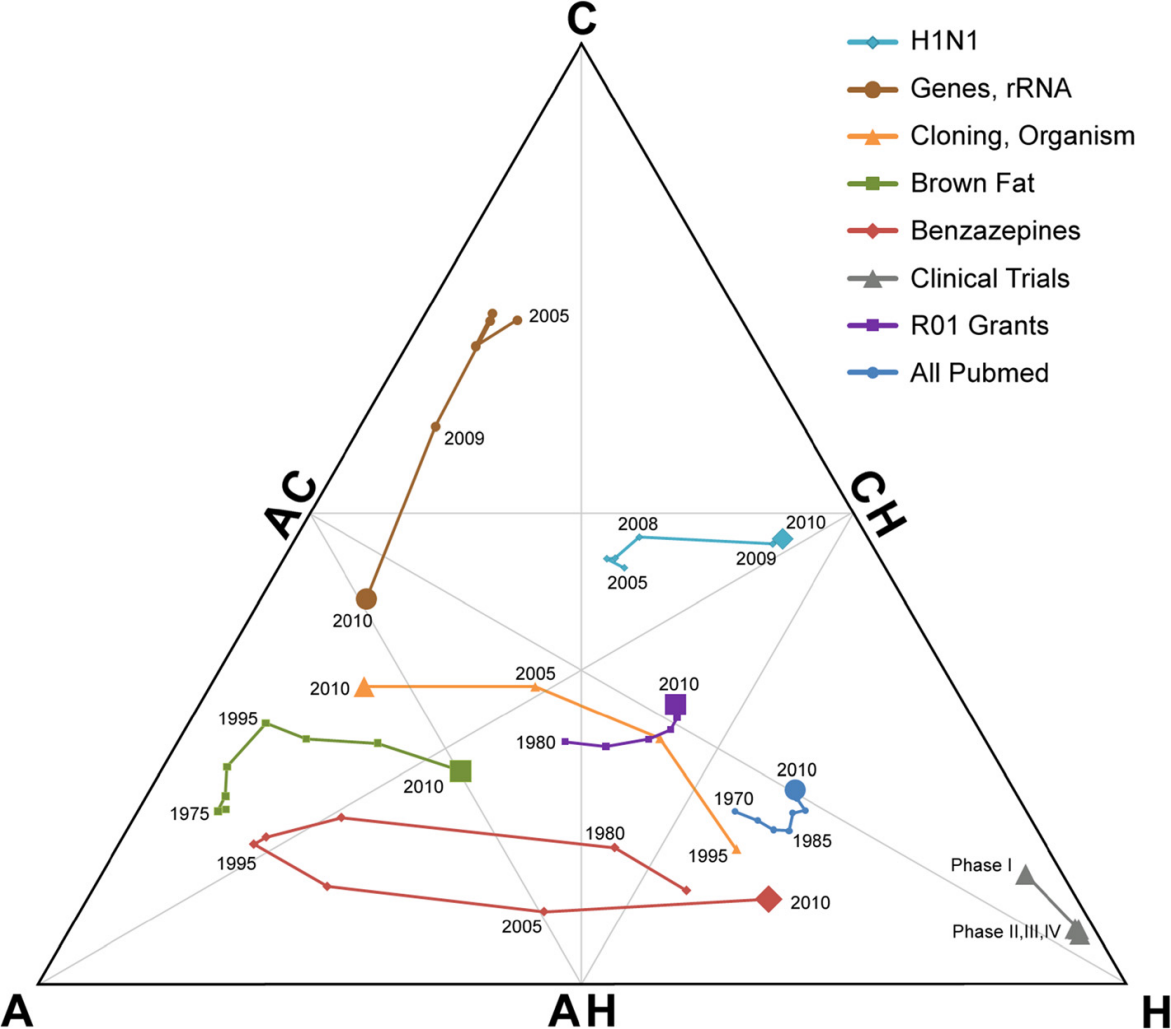
**Translation over time. **Each curve represents a different collection of articles: five based on MeSH descriptors, articles flagged in PubMed as being associated with a clinical trial, articles that cite R01 grants, and all articles in PubMed. The markers indicate different time points, with the largest marker indicating the position of the articles in 2010.

As knowledge in a research area evolves, its position in the Triangle of Biomedicine can move over time. Movement towards the H corner can be considered a transition in the focus of a research area from bench-to-bedside, and movement in the opposite direction indicates a return to basic science. Figure 2 shows some notable examples:

1) Articles with a MeSH descriptor “Adipose Tissue, Brown” (brown fat) were mostly focused on animal research until the late 1990s, when a number of related proteins were discovered and it was subsequently found that brown fat also exists in adult humans.

2) An almost immediate change in the focus of articles with a MeSH descriptor “Influenza A Virus, H1N1 Subtype” (swine flu) occurred with the 2009 pandemic of the virus in humans.

3) Articles with MeSH descriptors “Cloning, Organism” and “Genes, rRNA” have both moved in the direction of animal research in recent years.

4) The position of articles with a MeSH descriptor “Benzazepines” has swung in two directions with a surge in clinical trials during the early 1980s and again in the past five years, with a period of mostly animal research in the middle.

5) Not surprisingly, articles flagged in PubMed as Phase I clinical trials are near the H corner, and Phase II, III, and IV trials are progressively closer.

6) The publications that cite NIH R01 grant numbers cover a wide range of topics, and therefore are near the ACH point, though there has been movement towards the CH point over time.

7) PubMed as a whole has changed relatively little in the past 30 years, with a large percentage of its articles consistently in the H category.

### Translation occurs through incremental steps rather than giant leaps

Narin showed that articles in one research level primarily cite other articles in the same research level. Less common were citations in adjacent research levels, and only rarely did an article cite another that is two or three levels away [8]. In other words, the flow of knowledge typically did not go from basic research directly to clinical observation. Rather, it slowly passed through each of the research levels along the path towards translation.

**Figure 3:**
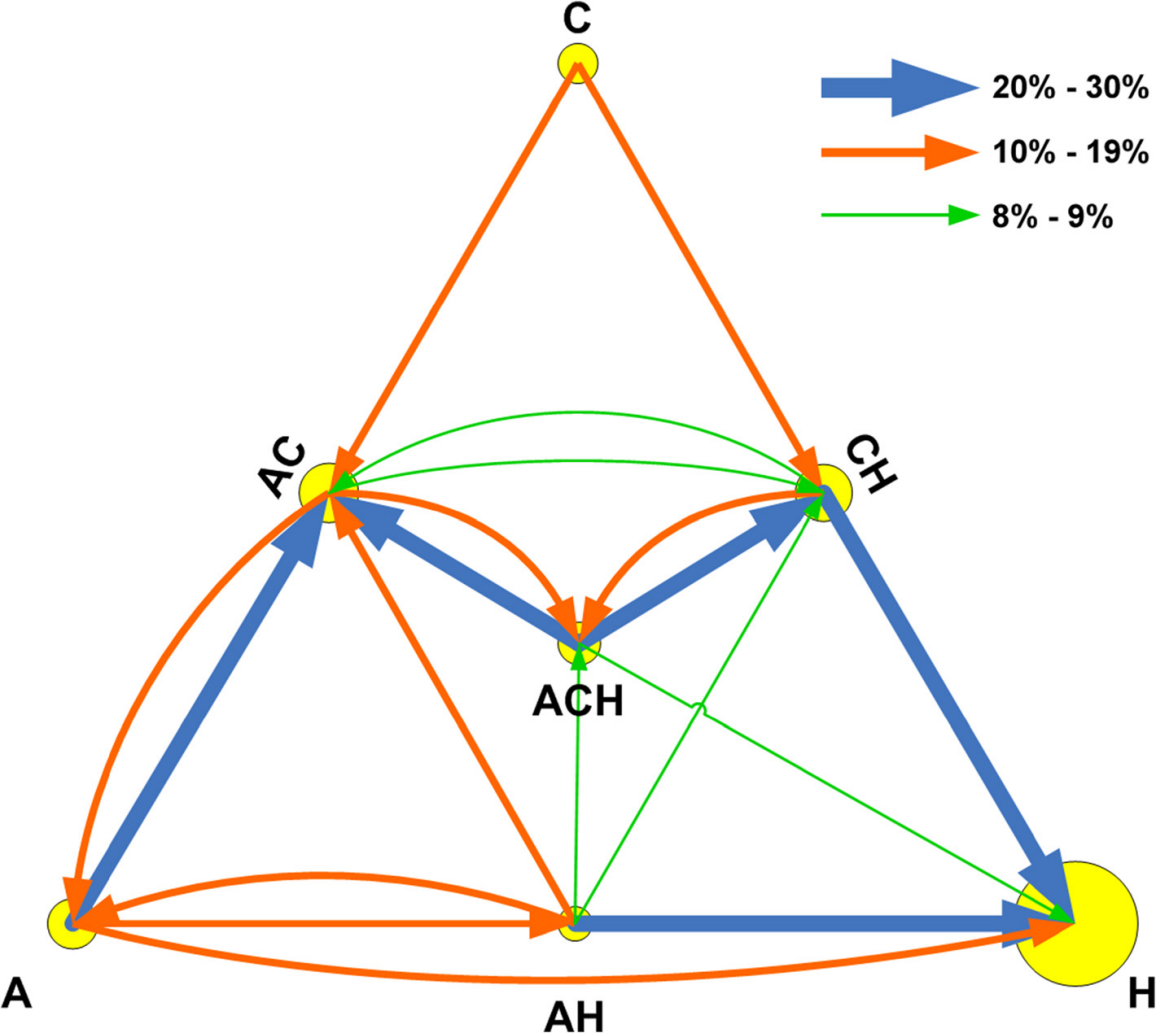
**Information flow between categories as measured by corrected citation counts.** Arrow size represents the percentage of citations of the category at the tail of the arrow. Circle area is proportional to the number of articles in that category.

Figure 3 shows a similar pattern of information flow within the Triangle of Biomedicine. In most cases, articles in a particular A-C-H category cite other articles within the same category. However, it is much more likely that an article will cite articles in adjacent categories than in ones on the opposite side of the triangle. Of all citations, 54% are articles citing articles in the same category, 36% are articles citing articles in adjacent categories (e.g., H citing AH, AC citing ACH, or AH citing A), and only 10% are articles citing articles in opposite categories (e.g., H citing A, C citing AH, or AC citing H). In other words, basic science research (A, AC, and C) rarely translates directly to H articles. Instead, it first passes through an intermediate stage (AH, ACH, and CH).

### Translation takes many years

The amount of time from a basic science discovery to a clinical intervention, the “translation lag”, can be many years [23,24]. Contopoulos-Ioannidis found that only 25% of high impact basic science articles that had clear therapeutic or preventative potential actually resulted in a clinical trial after 20 years; and, when translation occurs, it takes a median of 24 years from the initial basic science discovery until the first highly cited human study [25,26].

We use the concept of citation “generations” to measure the translation lag of an article. If one article cites a second article, which in turn cites a third article, then the second article is one citation generation from the first, and the third is two citation generations from the first. We assume that in order for an article to have clinical impact, it must reach an H article after some number of citation generations. While this does not guarantee that translation to clinical practice will occur, it gives us a lower bound on the amount of time it will take if it does. From this assumption, we define the following metrics:

1) Translational Fraction (TF) is the fraction of articles in a category that after some number of citation generations eventually reaches an H article.

2) Translational Distance (TD) of an article is the minimum number of citation generations needed to reach an H article. The TD of a collection of articles is the mean TD of the individual articles.

3) Translational Years (TY) of an article is the number of years it took to reach an H article. The TY of a collection of articles is the mean TY of the individual articles.

4) Translational Closeness (TC) is the average of the inverse of the TD of each article, including articles that have not yet translated (which have an inverse TD of zero). If TC = 1, then all articles are cited by H articles in exactly one generation; if TC = 0, then no articles have translated.

Table 1c lists the TF, TD, TY, and TC of each category. A, C, and AC articles take more citation generations and more time to reach H articles than AH, CH, and ACH articles, which is consistent with the results from the previous section. C articles require the most generations (TD = 3.08), though A articles require the longest time (TY = 10.40 years). Even H articles take 5.69 years, on average, before being cited by another H article.

The colors in Figure 1 indicate the average TD of different disciplines. There is a clear relationship between the TD and the position along the Translational Axis. For example, Nursing consists of mostly H articles (TI = 0.98), and nearly every article, when cited, is cited by an H article (TD = 1.09). Allergy and Immunology is near the ACH point (TI = 0.02) and requires an additional citation generation to reach an H article (TD = 2.29). Botany is furthest from the H point (TI = -0.46) and requires almost four citation generations (TD = 3.88).

### Training more physician-scientists could accelerate translation

Physician-scientists are essential in bridging the gap between basic research and clinical medicine and reducing the time to translation [24,27,28]. Zemlo notes that investigators with combined MD-PhD degrees play a particularly pivotal role in translation--they represent just 2.5% of medical school graduates each year, but have a third of the NIH grants going to physician-scientists [27].

**Figure 4:**
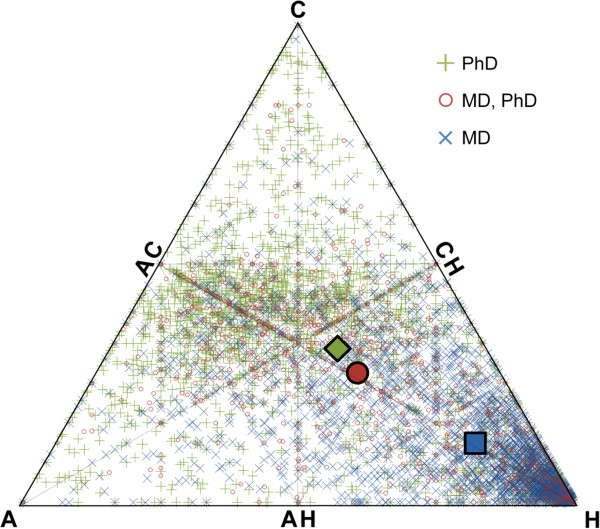
**Plots of all faculty from Harvard Medical School, separated by degree. **The large green diamond is the average position of faculty with PhD degrees; the large red circle is the average position of faculty with MD, PhD degrees; and the large blue square is the average position of faculty with MD degrees.

**Figure 5:**
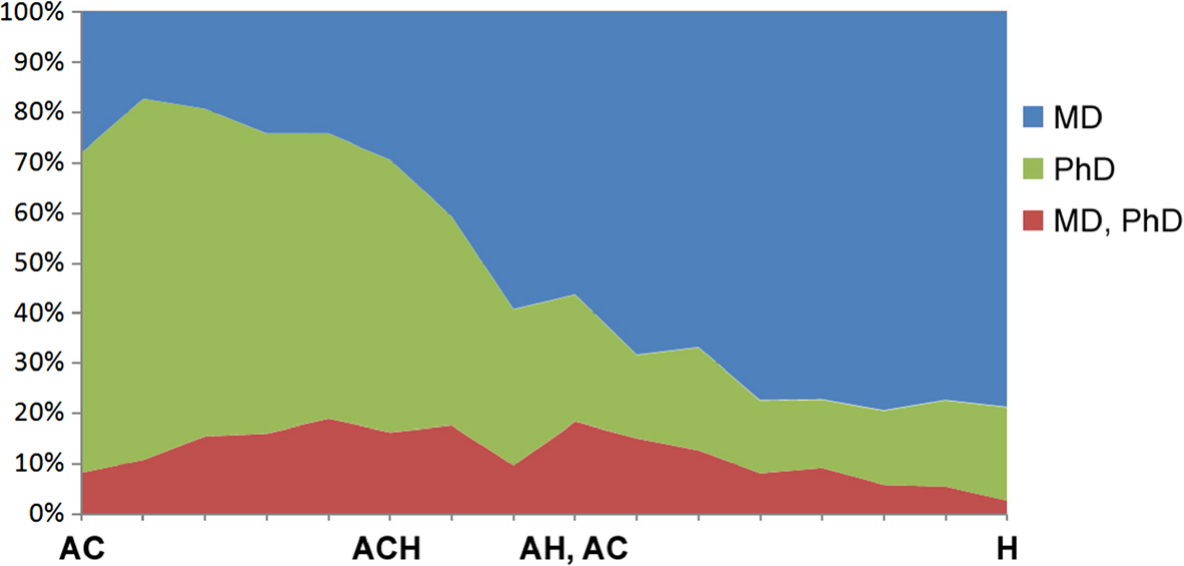
The percentage of faculty with different degree types along the AC-H translational axis.

Figures 4 and 5 plot all faculty from Harvard Medical School, separated by degree (PhD; MD-PhD; and MD), onto the Triangle of Biomedicine based on the articles they have published. The majority of faculty lie close to the Translational Axis—the line connecting AC and H, with fewer near the A and C points. Most faculty near H (TI = 1) have MD degrees, most faculty near AC (TI = -0.5) have PhD degrees, and faculty with both MD and PhD degrees are most likely to be near the ACH point (TI = 0). Thus, while there are many paths towards becoming an effective physician-scientist and translational researcher, faculty with MD-PhD degrees are most active at the points in the Triangle of Biomedicine though which translation typically occurs. Though beyond the scope of this study, one can also imagine performing similar analysis on groups of investigators, rather than at the individual person level, to determine if there are types of transdisciplinary teams that are likely to perform translational research.

## Discussion

Although the Triangle of Biomedicine is not meant to replace the traditional qualitative definitions of T1-T4 translational research [7], it provides a quantitative technique to measure translation and to determine how long it takes. This is important to policy makers in evaluating the impact of the large investments being made to accelerate translation. The Triangle of Biomedicine also provides a simple visual way of depicting this impact, which can be far more powerful than numbers alone.

As with other bibliometric techniques, it is important not to overgeneralize metrics. The position of a broad discipline on the Triangle of Biomedicine simply represents the average of thousands of publications. Predicting the potential impact of a specific research area or an individual article or scientist requires far more information; though, this comes with its own limitations. For example, a multidimensional scoring system has been developed to assess the “translatability” of drug development projects [29,30]. This may indeed be a superior method, but it requires manual review of the literature and therefore might not be scalable. Fontelo identified 59 words and phrases, which when present in the titles or abstracts of articles, suggest that the article is translational [31]. However, that is an all-or-nothing approach, which does not take into account the full spectrum from basic research to clinical medicine.

This work is limited in several ways. It takes at least a year for most articles to be assigned MeSH descriptors. During that time the articles cannot be classified using the method described in this paper. Also, our classification method is based on a somewhat arbitrary set of MeSH descriptors—different descriptors could have been used to map articles to A-C-H categories. However, the ones we used seemed intuitive and they produced results that were consistent with Narin’s classification schemes. Finally, any metric based on citation analysis is dependent on the particular citation database used, and there are significant differences among the leading databases [22]. In this study, we used citations in PubMed that are derived from PubMed Central because they are freely available in their entirety, and therefore our method can be used without subscriptions to commercial citation databases, such as Scopus and Web of Science, which are cost-prohibitive to most people. However, because these commercial databases have a greater number of citations and index different journals than PubMed, they might show shorter or alternative paths towards translation (i.e., fewer citation generations or less time). Though, as described in our Methods, there is evidence that suggests these differences might be relatively small. Selecting the best citation database for identifying translational research is a topic for future research.

Another area of future research could attempt to identify a subset of H articles that truly reflect changes in health practice and create a separate category P for these articles. This might be possible, for example, by using Khoury’s approach of using PubMed’s “publication type” categorization of each article to select for those that are clinical trials or practice guidelines [7]. This could be visualized in the Triangle of Biomedicine by moving H articles to the center of the triangle and placing P articles in the lower-right corner, thereby highlighting research that has translated beyond H into health practice.

## Conclusions

The Triangle of Biomedicine is a novel way to identify translational science and track changes over time. This is important to policy makers in evaluating the impact of the large investments being made to accelerate translation. As with any metric, its limitations and potential biases should always be kept in mind. As a result, it should be used to supplement rather than replace alternative methods of measuring or defining translational science. What is unique, though, to the Triangle of Biomedicine, is its simple visual way of depicting translation, which can be far more powerful to policy makers than numbers alone.

## Competing interests

The author declares that he has no competing interests.
